# Colonization of HIV-Infected Children with Methicillin-Resistant *Staphylococcus aureus*

**DOI:** 10.3390/pathogens8010035

**Published:** 2019-03-17

**Authors:** Eric S. Donkor, Fleischer C. N. Kotey, Nicholas T. K. D. Dayie, Samuel Duodu, Patience B. Tetteh-Quarcoo, Mary-Magdalene Osei, Edem M. A. Tette

**Affiliations:** 1 Department of Medical Microbiology, School of Biomedical and Allied Health Sciences, University of Ghana, P. O. Box KB 143, Korle Bu, Accra, Ghana; fleischarles@gmail.com or fcnkotey@flerholiferesearch.com (F.C.N.K.); nicholasdayie@yahoo.com (N.T.K.D.D.); patborket2002@yahoo.com (P.B.T.-Q.); m4maryosei@yahoo.com (M.-M.O.); 2 West African Centre for Cell Biology of Infectious Pathogens, University of Ghana, P. O. Box LG 54, Legon, Accra, Ghana; saduodu@ug.edu.gh; 3 FleRhoLife Research Consult, P. O. Box TS 853, Teshie, Accra, Ghana; 4 Department of Biochemistry, Cell and Molecular Biology, University of Ghana, P. O. Box LG 54, Legon, Accra, Ghana; 5 Department of Community Health, School of Public Health, University of Ghana, P. O. Box LG 13, Legon, Accra, Ghana; edemenator@gmail.com

**Keywords:** multidrug resistant, *Staphylococcus aureus*, *mec*A, infection

## Abstract

Background: Methicillin-resistant *Staphylococcus aureus* (MRSA) poses a public health threat owing to its extensive resistance to antibiotics, association with persistent outbreaks, and markedly increased healthcare costs. Moreover, HIV-infected individuals are at a greater risk for colonization with MRSA, and may act as reservoirs for subsequent transmission to other individuals. In Ghana, little is known about MRSA in relation to at-risk populations, such as HIV-infected children. The aim of this study was to investigate nasal carriage of *S. aureus* and MRSA among HIV-infected children in Accra, including the prevalence, risk factors and antibiotic resistance. Methodology: The study was cross-sectional, and involved 107 children with HIV infection and an equal number of sex- and age group- matched apparently healthy controls recruited from the Princess Marie Louis Children’s Hospital in Accra. Nasal swab specimens were collected from the study participants and cultured for bacteria. *S. aureus* isolates were confirmed by the coagulase test while MRSA was confirmed by PCR of the *mec*A gene. Antimicrobial susceptibility testing of *S. aureus* isolates was done by the Kirby Bauer method. A structured questionnaire was used to collect data on demographic, household and clinical features of the study participants. A logistic regression analysis was performed to identify determinants of *S. aureus* and MRSA carriage among participants of both study groups. Results: The carriage prevalence of *S. aureus* and MRSA were 44.9% (48) and 5.6% (6), respectively, among the HIV-infected individuals, and the corresponding values within the control group were 23.4% (25) and 0.9% (1). There was a significant association between HIV infection and *S. aureus* colonization (*p <* 0.001), but not MRSA colonization (*p* = 0.055). The main predictor of *S. aureus* colonization in both study groups was absence of colonization with coagulase negative staphylococcus (*p* < 0.001). Furthermore, the main predictor of MRSA colonization was regular hand washing with soap (*p* = 0.043); this was observed among HIV-infected individuals but not the control group. The proportion of *S. aureus* isolates that were multidrug resistant was 62.3% (33/53) in the HIV-infected group and 80% (20/25) in the control group (*p* = 0.192). Conclusions: HIV infection is a risk factor for nasal colonization of *S. aureus* among children in Accra but may not be for MRSA. Both the HIV-infected and uninfected children are reservoirs of multidrug resistant *S. aureus*. Demographic, household and clinical features appear to have little or no relationship with *S. aureus* and MRSA colonization in the study children.

## 1. Introduction

*Staphylococcus aureus* (*S. aureus*) causes a wide array of clinically important infections in humans, including meningitis, septicaemia, pneumonia, endocarditis, and osteomyelitis [1]. Although the pathogen can be carried at several body sites, its ecological niche is the anterior nares of the nose [2]. Among individuals of the general population, some 50% are apparently resistant to *S. aureus* colonization [3,4,5,6]. In contrast, 20% of individuals are estimated to be persistent carriers, and the other 30% carry the pathogen intermittently [4,5,6]. 

Some strains of *S. aureus* are referred to as methicillin-resistant *Staphylococcus aureus* (MRSA) owing to their resistance to methicillin. They are additionally resistant to all beta-lactam antibiotics. The remnant of the *S. aureus* strains are sensitive to methicillin, and are collectively referred to as methicillin-susceptible *Staphylococcus aureus* (MSSA). Because MRSA strains are primarily key nosocomial pathogens, they are referred to as healthcare-associated MRSA (HA-MRSA) [7]. Besides HA-MRSA strains, MRSA strains that are transmitted in the community, referred to as community-associated MRSA (CA-MRSA), have been reported [8,9,10,11]. CA-MRSA infections could also be caused by livestock-associated MRSA (LA-MRSA) [12,13]. Livestock-associated MRSA is initially associated with livestock (such as pigs, cattle, and chicken) and differs genotypically from HA-MRSA and CA-MRSA [14,15]. Back in 1970, MRSA accounted for only 2% of *S. aureus* infections [16]. By 2006, the pathogen had spread rapidly, and had caused up to 70% of *S. aureus* infections [17,18,19,20,21]. In Europe, it is implicated in about 44% of all infections related to healthcare [22]. Worse yet, its infections result in extended periods of hospitalization and increased healthcare costs [23]. In the United States, for instance, the annual incidence of invasive MRSA infections is estimated to be 94,360, resulting in 18,650 deaths [24]. Also, hospital stays for MRSA infections cost $14,000, in comparison with $7600 for all other stays, with twice the length of hospitalization [24,25].

HIV-infected patients are at a greater risk for colonization with CA-MRSA [26,27,28]. Studies across different geographical areas have reported high MRSA carriage prevalence of up to 16% in HIV-infected individuals [29,30,31,32]. This is of major concern as people with HIV infection have an 18-fold increased risk of acquiring CA-MRSA infections [33]. Furthermore, MRSA-colonized individuals may act as reservoirs for subsequent transmission to other individuals [34], and the occurrence of MRSA in patients is a significant predictor of increased morbidity and mortality [35,36,37]. In Ghana, surveillance data have reported MRSA carriage prevalence of 0–15% [38], though this does not include information on HIV-infected individuals. Since 2012, there have been several outbreaks of MRSA in Ghana [39], and the public health threat and substantial untoward economic impact associated with this pathogen places it high on the agenda of public health issues in the country. Clearly, MRSA has received little attention in Ghana, and this is partly because the focus of attention seems to be more towards microbes with a greater mortality burden in the country such as *Streptococcus pneumoniae* and *Rotavirus* [40,41]. As part of the overall strategy in addressing the potential MRSA menace in Ghana, there is a need for surveillance of the pathogen among risk populations, such as individuals with an HIV infection. The aim of this study was to investigate MRSA colonization among HIV-infected and uninfected children in Accra, including the prevalence, risk factors, and antimicrobial resistance patterns. 

## 2. Methods

### 2.1. Study Site, Design, and Sampling

The study was carried out at the Princess Marie Louise Children’s Hospital (PML) in Accra, the capital city of Ghana. According to the Ghana Statistical Service (http://www.statsghana.gov.gh/), the city is inhabited by about two million people and there are eight government (public) or quasi government hospitals. The PML Children’s Hospital is the only public hospital in the Accra Metropolis dedicated solely to children, and has a bed capacity of 92. The hospital has an HIV clinic, which runs twice a week with an attendance of about 20 patients. The study was cross-sectional, and involved 107 children with HIV infection and an equal number of sex- and age group-matched apparently healthy HIV-uninfected children (control group) recruited between January and July, 2017. The HIV-infected participants were recruited from among HIV-infected children attending the outpatient HIV clinic of PML Children’s Hospital, whilst the control group was recruited from the environs of the hospital. All children less than 15 years of age were eligible for the study. However, children who were on admission, those who had been on antibiotics (other than cotrimoxazole) two weeks or earlier prior to sampling, as well as those whose HIV status could not be determined, were excluded from the study. The HIV status of the study participants was obtained from their clinical records. A structured questionnaire was used to collect data on risk factors for *S. aureus* carriage from the study participants. The questionnaire was divided into three parts, namely, demography, household characteristics, and clinical features, and the responses were obtained by interview and review of patients’ folders.

Anterior nasal swabs were collected from the study participants by a trained paediatrician after obtaining informed consent from their parents/guardians. For each participant, a sterile cotton swab was rotated five times in both anterior nares. The swab specimen was then placed in a vial containing 1 mL skim milk–tryptone–glucose–glycerin (STGG) medium and transported on ice within four hours to the research laboratory of the Department of Medical Microbiology, on the Korle Bu campus of the University of Ghana. The specimens were then vortexed for about two minutes and stored in a −80° centigrade freezer until needed.

### 2.2. Isolation and Identification of S. aureus and MRSA

The specimens were enriched in 5 mL of tryptic soy broth, and subcultured onto blood, chocolate and MacConkey agar plates. The blood and chocolate agar plates were incubated at 37 °C in 5% CO_2_, whereas the MacConkey agar plates were incubated at 37 °C aerobically. The plates were examined for growth after 18–24 h. Isolates of staphylococci were identified based on colonial morphology, Gram stain, and the catalase test. Staphylococcal isolates were subjected to the tube coagulase test to determine whether they were *S. aureus* (positive coagulase test) or coagulase negative staphylococci (CONS). The identified *S. aureus* isolates were screened for cefoxitin resistance by the Kirby Bauer disc diffusion method and inhibition zones of ≤22 mm were interpreted as cefoxitin-resistant, and identified as MRSA [42]. 

### 2.3. Antimicrobial Susceptibility Testing

Susceptibility of *S. aureus* to standard antimicrobials was tested by the Kirby Bauer method, using the following antimicrobials: tetracycline (30 µg), erythromycin (15 µg), gentamicin (10 µg), rifampicin (5 µg), cotrimoxazole (1.25 + 23.75 µg), penicillin (10 µg), clindamycin (2 µg), fusidic acid (10 µg), cefoxitin (30 µg), and linezolid (10 µg). Each test isolate was emulsified in peptone water to create a suspension with a turbidity similar to that of 0.5% McFarland standard, with the help of a nephelometer. A sterile cotton swab was dipped into the suspension, and the swab was pressed against the interior walls of the container to drain excess fluid. The resultant swab was thereafter swabbed evenly across the entire surface of a Mueller Hinton agar plate, in three different dimensions, to obtain a semi-confluent growth following incubation. The plates were incubated at 37 °C for 18–24 h, after which the zones of inhibitions around the antimicrobial discs were measured and interpreted according to the breakpoints of the Clinical and Laboratory Standards Institute [42]. 

### 2.4. Molecular Analysis

Extraction of genomic DNA was performed on overnight lysogenic broth cultures of the MRSA isolates and a positive control strain (*S. aureus* ATCC 25923) using the Zymo Research extraction kit (Zymo Research Corp., Irvine, USA), following the instructions of the manufacturer. For quality control purposes, 5 µL of the extracted DNA from each isolate was mixed with 2 µL of bromophenol blue gel loading buffer and run on a 1.2% agarose gel, and the bands visualized by UV illumination. Presence of the *mec*A and *nuc*A genes were determined by conventional PCR amplification using the extracted DNA samples as templates. The total reaction volume for the PCR was 50 uL, consisting of the genomic DNA (of final concentration 60 ng/µL), PCR water, primers (of final concentration 0.2 µM), Taq polymerase (of final concentration 1.25 U/µL), MgCl_2_ (of final concentration 2 mM), and deoxyribonucleotide triphosphates (dNTPs) (of final concentration 200 µM). RNase-Free Water was used as the negative control, and the amplicons were separated by a 1.2% agarose gel electrophoresis and visualized by UV illumination. For the *mec*A PCR, the primer sequences used were ATCGATGGTAAAGGTTGGC (forward primer) and AGTTCTGCAGTACCGGATTTGC (reverse primer), and the cycling conditions were: initial denaturation at 95 °C for 3 min; denaturation at 94 °C for 1 min; annealing at 55 °C for 30 s; extension at 72 °C for 1 min; final extension at 72 °C for 6 min. The total number of cycles was 33. Using the same cycling conditions as those used in the *mec*A PCR, a *nuc*A PCR was performed on all the DNA samples; the primer sequences used were GCGATTGATGGTGATACGGTT (forward primer) and AGCCAAGCCTTGACGAACTAAAGC (reverse primer).

### 2.5. Data Analysis

Data was entered into MS Excel and imported into STATA 14 (Strata Corp, College Station, TX, USA) for analysis. Descriptive analyses were performed on the study variables. Univariate associations were performed between *S. aureus*/MRSA colonization and demographic, clinical and household features: analysis of variance was used for numeric variables, whereas a chi-square test was used for categorical variables. A logistic regression model was used to analyze exposures associated with *S. aureus*/MRSA colonization, and the results were presented as Odds Ratios (OR), *p* values and Confidence Intervals (95% CI). Antibiogram and multidrug resistance of *S. aureus*, including MRSA isolates were computed; multidrug resistance was defined as resistance to three or more classes of antimicrobial agents.

### 2.6. Ethical Approval

The study was approved by the Ethical and Protocol Review Committee of the College of Health Sciences, University of Ghana, with protocol identification number “CHS-Et/M.3 – P 4.4/2016-2017”.

## 3. Results

### 3.1. Demographic, Household, and Clinical Features of the Study Participants

Demographic and household features of the two study groups (HIV-infected and HIV-uninfected children) are presented in Table 1. For each group, the males and females comprised 54.2 and 45.8%, respectively. The mean body mass index (BMI) of the HIV-infected and HIV-uninfected participants was 16.41 Kg/m^2^ and 17.66 Kg/m^2^, respectively. The mean age of the HIV-infected participants was 6.36 years, and for the HIV-uninfected participants, it was 6.32 years. With the exception of those aged less than 1 year who comprised 1.9% in each study group, the age distribution was similar for the rest of the age groups in both study groups, namely, 1–4 years (38.3% in the HIV-infected group and 37.4% in the HIV-uninfected group), 5–9 years (32.7% in the HIV-infected group and 36.4% in the HIV-uninfected group), and > 9 years (27.1% in the HIV-infected group and 24.3% in the HIV-uninfected group). For both study groups, a greater proportion of the participants were currently enrolled in school (74.8% for the HIV-infected group and 83.2% for the HIV-uninfected group), rarely washed their hands with soap (92.5% in the HIV-infected group and 95.3% in the HIV-uninfected group), and lived in compound houses (86% for the HIV-infected group and 84.1% for the HIV-uninfected group). The number of individuals per household, decreased across <5 persons (61.7% for the HIV-infected and 58.9% for the control group), 5–10 persons (35.5% for the HIV-infected and 40.2% for the control group), and 11–20 persons (2.8% for the HIV-infected and 0.9% for the control group), and less than 4% of the participants lived in households which had a health worker.

The clinical features of the study participants are presented in Table 2. In the HIV-infected group, a greater proportion of the participants were on co-trimoxazole prophylaxis (88.8%) and antiretroviral therapy (89.7%), and indicated that they practiced self-medication (58.9%, which is higher than the 41.1% recorded in the HIV-uninfected group). Also, a greater proportion of them had been hospitalized in the past year than those in the HIV-uninfected group (29.9% vs. 11.2%), with 68.9% of that proportion (as opposed to 100% in the HIV-uninfected group) being hospitalized once and 31.1% (as opposed to 0% in the HIV-uninfected group) being hospitalized twice. None of the participants in both groups had a chronic skin condition or a history of surgery. However, compared to the HIV-uninfected group, more participants in the HIV-infected group had a history of pneumonia (4.7% vs. 0.9%) and tuberculosis (6.5% vs. 0%).

### 3.2. Carriage Prevalence and Risk Factors of S. aureus and MRSA

Overall, *S. aureus* carriage occurred in 73 of the 214 study participants, which translates to a prevalence of 34.1% (95% CI: 28.1–40.7), while MRSA carriage occurred in 7, representing 3.3% (95% CI: 1.6–6.6). Among the HIV-infected individuals, carriage prevalence of *S. aureus* and MRSA were 44.9% (48) and 5.6% (6), respectively. Among the HIV uninfected individuals, carriage prevalence of *S. aureus* and MRSA were 23.4% (25) and 0.9% (1), respectively. There was a significant association between HIV infection and *S. aureus* colonization (OR = 2.67, *p* = 0.001), but not MRSA colonization (OR = 3.69, *p* = 0.055).

In the logistic regression analysis, the main predictor of *S. aureus* colonization was absence of colonization with coagulase negative staphylococcus (CONS), in both the HIV-infected and HIV-uninfected participants. Furthermore, the main predictor of MRSA colonization was regular hand washing with soap; this was observed among HIV-infected individuals, but not the control group. None of the demographic, household, or clinical features assessed showed significant association with *S. aureus* and MRSA colonization in either the HIV-infected or the control group. The risk factors for *S. aureus* and MRSA colonization are presented in Table 3.

Of the 7 cefoxitin-resistant *S. aureus* (MRSA) isolates, 5 (comprising 4 in the HIV-infected group and 1 in the control group) contained the *mec*A gene. The *nuc*A gene, however, was present in all the 7 MRSA isolates.

### 3.3. Antibiotic Resistance of S. aureus and MRSA Isolates

**Figure 1:**
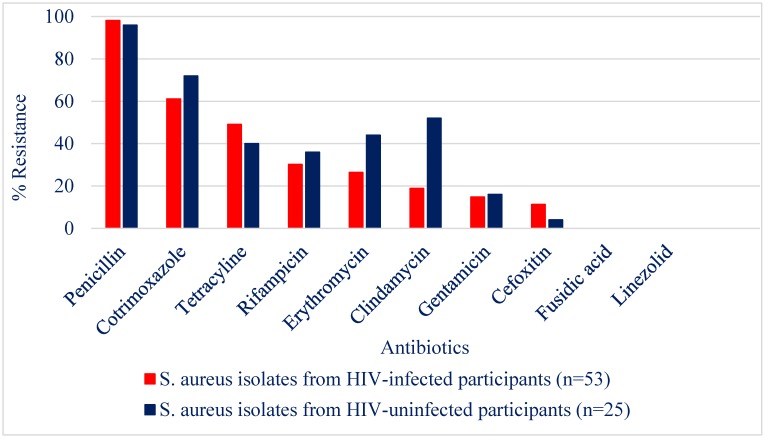
Antibiogram of *S. aureus* isolated from HIV-infected and uninfected children.

The highest prevalence of antibiotic resistance of *S. aureus* was observed for penicillin (98.1% in the HIV-infected group and 96% in the control group), followed by cotrimoxazole (61% in the HIV-infected group and 72% in the control group); no resistance was observed for fusidic acid and linezolid (Figure 1). Generally, there were no significant differences between HIV-infected children and the control group in *S. aureus* resistance of the various antibiotics tested with the exception of clindamycin (*p* = 0.003). The proportion of multidrug resistant *S. aureus* was 62.3% (33/53) in the HIV-infected group and 80% (20/25) in the control group (*p* = 0.192). As shown in Table 4, multidrug resistance of the *S. aureus* isolates from both HIV-infected and uninfected individuals involved a diverse combination of resistance to several antibiotics.

For both HIV-infected and uninfected children, *S. aureus* resistance was highest for penicillin and least for fusidic acid and linezolid. There was no significant difference in *S. aureus* resistance between the two study groups except for clindamycin (*p* = 0.003). The *S. aureus* isolates from HIV-infected children includes dual carriage in 5 children, hence the discrepancy between the number of isolates tested (53) and the carriage prevalence reported for this group (48/107).

## 4. Discussion

To the best of our knowledge, this study is the first case-control study on *S. aureus* and MRSA colonization related to HIV-infected people in Ghana. The results of this study indicate a significantly higher prevalence of *S. aureus* among the HIV-infected children (44.9%) compared to the control group (23.7%). The *S. aureus* prevalence among HIV-infected individuals in our sample is in stark contrast with the 8% prevalence reported in an earlier study involving HIV-infected individuals in Ghana by Egyir et al. [43]. It should be noted, however, that the participants in Egyir et al.’s study were adults and consisted of both inpatients and outpatients [43], whereas those in the current study were ambulatory children. Even though another earlier study conducted among HIV-infected children in Ghana also reported a relatively lower prevalence of 22.0%, it is noted that these researchers used swabs from the nasopharynx [44], which is not an ecological niche for *S*. *aureus*. That the source of swabs used to determine prevalence of *S. aureus* influences the level of prevalence is attested to by the findings of other researchers [32,45]. These researchers recorded approximately 2–3-fold higher prevalence of *S*. *aureus* from nasal specimens than from the perineal swabs or oral swabs. Our study is most comparable to the case-control study conducted in India by Kotpal et al. [46] who reported a 44% *S. aureus* nasal carriage prevalence among HIV-infected individuals; this prevalence was significantly higher than that observed in the control group in their study (44% vs. 24%; *p* = 0.035). 

The absence of statistical significance with regard to the difference in MRSA colonization between the HIV-infected and the control group is similar to the observation made in Nigeria by Olalekan et al. [47], whose higher MRSA prevalence among HIV-infected participants was not significantly different from that recorded within the control group (16% vs. 8%; *p =* 0.13). The observed MRSA nasal carriage prevalence among the HIV-infected children is higher than the 0% reported by Egyir et al. [43] among HIV-infected adults, and marginally higher than that reported in the nasopharynx of HIV-infected children (3.4%) by Sampane-Donkor et al. [44]. However, it is substantially lower than the 16.8% and 21% reported by Lemma et al. [32] in Ethiopia and Heysell et al. [48] in South Africa, respectively. Almost half (42%) of the participants in the study of Lemma et al. [32] were designated to be at WHO stage III and IV of HIV infection, which is an indication of advanced infection. The participants in the study of Heysell et al. [48] were residents of an HIV endemic region, had advanced HIV infection (median CD4+ count = 37 cells/mm^3^), were co-infected with TB, and were also hospitalized. Notably, hospitalization may facilitate the spread of MRSA colonization via contaminated surfaces or direct contact with patients/healthcare providers carrying MRSA [49]. These may account for why the two studies (Heysell et al. [48]; Lemma et al. [32]) recorded a markedly higher MRSA prevalence than the current study.

It has been reported in earlier studies that HIV-infected individuals are predisposed to MRSA colonization [26,27,28,50,51]. This study found no such association. The reason for this observation is unclear, but could be multifactorial. The study participants were in steady state, and their health had not deteriorated to an extent that would require hospitalization; in fact, their mean CD4+ count was 731.2 cell/mm^3^, 89.7% of the participants were on antiretroviral therapy, and only 29.9% of them had been hospitalized in the year prior to the commencement of the study. Antiretroviral therapy has been demonstrated to reverse some of the immunologic abnormalities that predispose HIV-infected individuals to colonization with MRSA [52]. Moreover, lower CD4+ count, history of antibiotic use in the past three months, prior hospitalization, and disease condition have all been linked to a higher rate of MRSA colonization [49,50,53,54].

Our study indicates that colonization with CONS appears to be protective of *S. aureus* colonization in both HIV-infected and HIV-uninfected children. This finding is consistent with the report of Paharik et al. [55] which showed that CONS prevent *S. aureus* colonization by inhibiting *agr* quorum sensing in *S. aureus* using its autoinducing peptide. With regard to MRSA colonization, none of the variables were demonstrated to be a predictor of colonization among the HIV-uninfected individuals. This was expected, given that only one HIV-uninfected participant was MRSA-colonized. Regular hand washing was shown to be a significant predictor of MRSA colonization among the HIV-infected participants, which is interesting given that good hygiene practices have been promoted as a means of controlling the spread of several pathogens [56,57,58]. This finding that regular hand washing with soap is a determinant of MRSA colonization, is supported by a recent study by Syed et al. [59]. In that study, triclosan, a biocidal component of soaps and many skin care products, was shown to be commonly present in nasal secretions, and to facilitate *S. aureus* binding with nasal proteins, such as keratin, fibronectin, and collagen. Consequently, hand washing with soaps that contain triclosan could potentially increase the risk of colonization with *S. aureus* and MRSA. In Ghana, there is the need for further studies on this, including soap screening for triclosan, and evaluating the effect of triclosan containing soaps on MRSA carriage through a longitudinal study approach.

Though high prevalence of resistance was observed for most of the antibiotics tested, very low prevalence of resistance was recorded for fusidic acid and linezolid among the *S. aureus* isolates in both the HIV-infected and the control groups. The absence of fusidic acid resistance recorded in this study was contrastingly lower than that reported in a cohort of HIV-infected individuals (10%) in Ghana [43] and another study in the general population (12%) [60], but consistent with the low rates of 0–2.4% reported by Dekker et al. [61], Egyir et al. [62], and Egyir et al. [63], also in Ghana. Moreover, the prevalence of linezolid resistance recorded for *S. aureus* in this study is consistent with what have been reported in other studies [41,60,61]. These two antibiotics are not widely available in the country, are quite expensive, and are rarely used or abused, possibly accounting for the low prevalence of resistance observed. It is important to note that two of the cefoxitin-resistant *S. aureus* isolates tested negative for the *mec*A gene, which predominantly mediates methicillin resistance in *S. aureus* [64]. However, these isolates were positive for the *nuc*A gene, confirming them to be *S. aureus* [65]. Hence, methicillin resistance in the two isolates may be mediated by the novel methicillin resistance gene, the *mec*C gene, rather than the initially reported *mec*A gene. In fact, *mec*C-mediated methicillin resistance is emerging, and has been widely reported [66].

The prevalence of MDR *S. aureus* in this study (62.3% among HIV-infected children and 80% among the control group) is within the range of 30–84.6% reported in other HIV-infected cohorts [43], but higher than what has been recorded by studies involving the general population (6–35.7%) in Ghana [60,61,62,63]. The prevalence of MDR observed in the current study is alarming, though not surprising. In Ghana and many other developing countries, antibiotics may be acquired without any prescriptions [67,68,69]. Also, self-medication with antibiotics is common practice, with the prevalence reportedly being 70–75% [68,70]. In the study by Donkor et al. [70], they reported that about 50% of the study respondents did not complete an antibiotic course. Furthermore, antibiotic misuse is high in animal husbandry in Ghana [71], and occurs at the hospital level as well [72]. These factors contribute to the high prevalence of MDR observed in the current study and other studies in Ghana [40,44,60,61,73]. The high prevalence and rates of self-medication among the general public in Ghana [68,70], could explain why we did not observe a significantly higher prevalence of cotrimoxazole resistance among the HIV-infected children compared to the control group, though the antibiotic is used in prophylaxis among this population. The significantly higher prevalence of clindamycin resistance among the control group in this study is difficult to explain. In Ghana, clindamycin is hardly used in self-medication, and the antibiotic is not specifically used for HIV-patients like in the case of cotrimoxazole.

Generally, data from the current study indicate that prevalence of MRSA carriage among children in the study area is generally low (< 4%), which is corroborated by other studies in Ghana [38,74,75]. The low MRSA carriage prevalence reported in Ghana most probably accounts for the very low incidence of invasive MRSA infections reported by some studies in the country [38,76]. Based on our data, we hypothesize a low incidence of invasive MRSA infections among HIV-infected children in Ghana. 

The study concludes that HIV infection is a risk factor for *S. aureus* colonization among children in Accra, but may not be for MRSA colonization. Also, absence of colonization with coagulase negative staphylococcus is a risk factor for *S. aureus* colonization, regardless of HIV infection status. Regular hand washing may be significantly associated with MRSA colonization, which could be due to triclosan in soaps/detergents used for hand washing. Finally, both HIV-infected and uninfected children are reservoirs of multidrug resistant *S. aureus*, which are entirely susceptible to fusidic acid and linezolid.

## Figures and Tables

**Table 1:** Demographic and household characteristics of the study participants.

Demographic and Household Characteristics	HIV-Infected		HIV-Uninfected	
	Number	%	Number	%
Age				
<1 year	2	1.9	2	1.9
1–4 years	41	38.3	40	37.4
5–9 years	35	32.7	39	36.4
>9 years	29	27.1	26	24.3
Gender				
Male	58	54.2	58	54.2
Female	49	45.8	49	45.8
Current school enrolment				
Yes	80	74.8	89	83.2
No	27	25.2	18	16.8
Type of residence				
Self-contained	15	14	17	15.9
Compound	92	86	90	84.1
Number of individuals in household				
<5	66	61.7	63	58.9
5–10 persons	38	35.5	43	40.2
11–20 persons	3	2.8	1	0.9
Presence of health worker in household				
Yes	4	3.7	3	2.8
No	103	96.3	104	97.2
Hand washing with soap				
Rarely	99	92.5	102	95.3
Often	8	7.5	5	4.7

BMI (HIV-infected) $\left(\bar{X},\;SD\right)$ = 16.41, 9.06 Kg/m^2^; BMI (HIV-uninfected) $\left(\bar{X},\;SD\right)$ = 17.66, 3.87 Kg/m^2^.

**Table 2:** Clinical features of the study participants.

Clinical Features	HIV-Infected		HIV-Uninfected	
	Number	%	Number	%
Self-reported self-medication				
Yes	63	58.9	44	41.1
No	44	41.1	63	58.9
Cotrimoxazole prophylaxis				
Yes	95	88.8	0	0
No	12	11.2	107	100
Antiretroviral therapy				
Yes	96	89.7	0	0
No	11	10.3	107	100
History of hospitalization in the past year				
Yes	32	29.9	12	11.2
No	75	70.1	95	88.8
Frequency of hospitalization in the past year				
0	75	70.1	95	88.8
1	22	20.6	12	11.2
2	10	9.3	0	0
Chronic skin condition				
Yes	0	0	0	0
No	107	100	107	100
History of pneumonia				
Yes	5	4.7	1	0.9
No	102	95.3	106	99.1
History of TB				
Yes	7	6.5	0	0
No	100	93.5	107	100
History of surgery				
Yes	0	0	0	0
No	107	100	107	100

Mean Cluster of Differentiation 4 (CD4+) count of HIV-infected participants = 731.20 ± 641.98 cells/mm^3.^

**Table 3:** Risk factors for *S. aureus* and methicillin-resistant *Staphylococcus aureus* (MRSA) colonization.

Risk Factor	HIV-Infected		HIV-Uninfected	
	OR (95% CI)	*p* Value	OR (95% CI)	*p* Value
Colonization with coagulase negative staphylococcus (CONS) ^*^	0.078 (0.028–0.217)	<0.001	0.038 (0.008–0.174)	<0.001
Regular hand washing ^+^	6.462 (1.06–39.395)	0.043	N/A	N/A

* associated with *S. aureus* colonization; ^+^ associated with MRSA colonization; N/A = Not applicable.

**Table 4:** Antibiotic resistance patterns of *S. aureus* isolates.

HIV-Infected Group		Control Group	
Resistance Pattern	n	Resistance Pattern	n
Pen	6	Pen	3
Tet-Pen	7	Pen-Cln	2
Tet-Cot	1	Cot-Pen-Cln	1
Cot-Pen	6	Tet-Cot-Pen	1
Rif-Pen	2	Gen-Cot-Pen	1
Tet-Cot-Pen	9	Ery-Cot-Cln	1
Ery-Cot-Pen	1	Ery-Rif-Pen-Cln	1
Tet-Pen-Cln	1	Ery-Cot-Pen-Cln	1
Rif-Cot-Pen	1	Ery-Gen-Cot-Pen	1
Rif-Pen-Cln	1	Tet-Ery-Cot-Pen	1
Ery-Rif-Cot-Pen	1	Tet-Cot-Pen-Cln	1
Rif-Cot-Pen-Cln	1	Tet-Gen-Rif-Pen	1
Rif-Pen-Cln-Cef	2	Tet-Rif-Cot-Pen	1
Gen-Cot-Pen-Cef	1	Tet-Ery-Cot-Pen	1
Ery-Cot-Pen-Cef	1	Rif-Cot-Pen-Cln	1
Tet-Ery-Cot-Pen	1	Tet-Ery-Cot-Pen	1
Tet-Rif-Cot-Pen-Cln	1	Gen-Rif-Cot-Pen	1
Tet-Gen-Rif-Cot-Pen	1	Ery-Rif-Cot-Pen-Cln	2
Tet-Ery-Gen-Cot-Pen	2	Tet-Cot-Pen-Cln-Cef	1
Tet-Ery-Cot-Pen-Cln	1	Tet-Ery-Rif-Cot-Pen-Cln	2
Tet-Ery-Rif-Cot-Pen	1		
Ery-Gen-Rif-Cot-Pen	1		
Ery-Rif-Pen-Cln-Cef	1		
Ery-Gen-Rif-Cot-Pen-Cln	1		
Tet-Ery-Gen-Rif-Cot-Pen	1		
Tet-Ery-Gen-Rif-Cot-Pen-Cln-Cef	1		

Pen = Penicillin; Tet = Tetracycline; Cot = Cotrimoxazole; Rif = Rifampicin; Ery = Erythromycin; Cln = Clindamycin; Gen = Gentamicin; Cef = Cefoxitin; n = number of resistant isolates.
